# Prophylactic endoluminal vacuum-assisted therapy in high-risk ultra-low rectal anastomoses: a retrospective comparative study of feasibility and clinical outcomes

**DOI:** 10.1186/s12893-026-03771-w

**Published:** 2026-08-27

**Authors:** Nour Alkhanji, Eslam Elmaghraby, Saleh Lahes, Mohammed Abdulla, Wolfram Trudo Kneofel, Nadja C Lehwald-Tywuschik

**Affiliations:** 1 Department of Surgery, University Hospital Knappschaft Kliniken, Ruhr University Bochum, Bochum, Germany; 2 Department of Surgery, University Hospital Dusseldorf, Dusseldorf, Germany

## Abstract

**Background:**

Anastomotic leakage (AL) remains a major cause of morbidity and mortality after ultra-low anterior resection (uLAR) for rectal cancer, when the anastomosis is located ≤ 5 cm from the anal verge, in patients with additionally risk factors. This study proposes the prophylactic endoluminal vacuum-assisted therapy (pEVAT) as a feasible strategy that may reduce AL incidence and promote healing in this subgroup of patients.

**Methods:**

Thirty-three patients multiple established risk factors for AL who underwent uLAR with a diverting stoma were retrospectively analyzed. Thirteen patients received pEVAT intraoperatively using a polyurethane sponge, while twenty patients served as the control group. The main outcomes included AL rate, secondary outcomes were anastomotic stenosis, other postoperative complications, length of hospital stay as well as stoma reversal.

**Results:**

Anastomotic leakage occurred in 7.7% of patients receiving pEVAT versus 30% in the control group. Unadjusted risk estimation demonstrated lower odds of leakage in the pEVAT group, but the difference was not statistically significant. Anastomotic stenosis was also observed in 7.7% of the pEVAT group compared to 35% of controls. The stoma reversal rate was higher in the pEVAT group (76.9% vs. 55%). Mean hospital stay was shorter (26 vs. 36 days). No mortality occurred in the pEVAT group, and complications were limited.

**Conclusion:**

Prophylactic EVAT is a feasible and safe approach that may have a protective role against anastomotic complications in selected high-risk patients undergoing uLAR. Larger, prospective, and ideally randomized studies are required to clarify its clinical effect in this subgroup.

## Introduction

Anastomotic leakage following rectal resection remains one of the most serious complications in colorectal surgery. Despite improvements in surgical techniques and perioperative management, the rate of an anastomotic leak (AL) after low anterior rectal resection (LAR) defined as an anastomosis located ≤ 5 cm from the anal verge, remains high and a six-fold increase in the risk of AL has been reported [1–3] there are many well known risk factors for AL such as advanced age, high ASA score, immunosuppression, radiation, and technical factors such as tension at the anastomotic site or stapler failure [1, 4–6].

Traditional strategies such protective diverting stomas primarily mitigate the clinical consequences of leakage rather than prevent its occurrence [7, 8]. Endoluminal vacuum-assisted therapy (EVAT) has been established as an effective treatment modality for clinically manifest anastomotic leakage [9–13]. However, its use as a prophylactic method (pEVAT) has not yet been widely studied. In 2021, we successfully published the first series of patients who received pEVAT for different underlying pathologies [14]. The aim of the present study was to evaluate the feasibility and clinical outcomes of prophylactic pEVAT in a selected cohort of high-risk patients undergoing ultra-low rectal anastomosis for colorectal cancer, compared with a contemporaneous control group managed without prophylactic EVAT.

## Methods

### Study design and ethics approval

This retrospective single-centre comparative study was conducted at a tertiary referral hospital (Department of Surgery, Heinrich-Heine-University Hospital Duesseldorf, Germany). Although the author group includes collaborators from two academic institutions, all patients included in the analysis had colorectal cancer and were treated at the same centre. The collaboration was mainly regarding data-analysis and interpretation. Prophylactic endoluminal vacuum-assisted therapy (pEVAT) was applied as part of clinical care based on intraoperative assessment and surgeon judgment in selected high-risk cases and was not introduced within a predefined research protocol. Ethics committee approval was obtained prior to data collection and analysis for this retrospective study.

### Patient selection

Patients undergoing ultra-low anterior resection (uLAR) with colorectal or coloanal anastomosis located ≤ 5 cm from the anal verge between 2016 and 2020 were eligible for inclusion. pEVAT was applied in patients in whom the anastomosis was considered to be at particularly high risk for leakage based on intraoperative judgment of the operating surgeon and cumulative risk factors. A contemporaneous control group consisted of patients undergoing uLAR for the same pathology during the same period who did not receive pEVAT.

### Risk factors for anastomotic leakage

Risk factors for anastomotic leakage were predefined based on existing literature and are summarized in Table 1. These included patient-related and procedure-related variables. Immunosuppression was defined as chronic systemic corticosteroid therapy exceeding 10 mg/day methylprednisolone equivalent or other immunosuppressive agents. Chemotherapy was analysed separately and was not subsumed under immunosuppression.

**Table 1:** Patients’ characteristics and risk factors in both groups

Patient characteristics	pEVAT *n* = 13	Control *n* = 20	*P*-value
Age years (mean ± SD)	64.9 ± 13.5	65.6 ± 13	
Sex n (%)			
male	9 (69.2)	13 (62.5)	
female	4 (30.8)	7 (37.5)	
BMI kg/m² (mean ± SD)	26.2 ± 3.7	25.3 ± 4.8	
Diverting stoma n (%)			
Jejunostomy	2 (15.4)	1 (5)	
Ileostomy	4 (30.8)	3 (15)	
Colostomy	7 (53.8)	16 (80)	
Anastomosis level cm (mean ± SD)	3.4 ± 0.9	3.5 ± 1.1	
Stapled anastomosis n (%)	11 (84.6)	16 (80)	
Advanced age> 65years n (%)	7 (53.8)	12 (60)	*0.72*
ASA-score ≥ 3 n (%)	7 (53.8)	8 (40)	*0.43*
Cachexia n (%)	0	1 (5)	*0.42*
Overweight n (%)	7 (53.8)	10 (50)	*0.82*
Cardiovascular diseases n (%)	6 (46.2)	3 (15)	*0.05*
Diabetes mellitus n (%)	3 (23.1)	4 (20)	*0.83*
Radiotherapy	6 (46.2)	12 (60)	*0.43*
CTX or HIPEC n (%)	5 (38.5)	10 (50	*0.51*
Immune suppression n (%)	1 (7.7)	1 (5)	*0.75*
Duration of surgery > 300 min. n (%)	10 (76.9)	16 (80)	*0.83*
Acute infection or sepsis n (%)	0	1 (5)	*0.41*
Intraoperative blood Transfusion n (%)	5 (38.5)	9 (45)	*0.71*
Frozen Pelvis n (%)	4 (30.8)	1 (5)	*0.04*
Dysfunction of the stapler	3 (23.1)	0	*0.02*

*pEVAT* Prophylactic endoluminal vacuum-assisted therapy, *SD* Standard deviation, *BMI* Body-mass-index, *ASA* American society of Anesthesiologists, *CTX* Chemotherapy, *HIPEC* Hyperthermic, intraperitoneal chemotherapy

### Anastomotic technique and measurement

All anastomoses were constructed in an end-to-end fashion. In patients undergoing stapled anastomosis, the distal rectum was transected under direct visualization using scissors rather than a linear stapler. The open rectal or anal stump was subsequently closed using a purse-string suture. Following this, the circular stapler was introduced transanally, and the anastomosis was completed in a single-stapling fashion. This approach was used to avoid the formation of “dog ears” typically associated with conventional double-stapled anastomoses. Hand-sewn anastomoses were performed transanally using interrupted sutures under direct visualization with a Lone Star^®^ retractor system.

Anastomotic height was measured as the distance between the anastomosis and the anocutaneous line using a rigid proctoscope. Individual measurements were recorded in whole centimetres.

### pEVAT technique

The Endo-Sponge^®^ system (B. Braun, Melsungen, Germany) connected to a Redyrob^®^ Trans Plus suction device was used for prophylactic therapy. The sponge was placed transanally intraoperatively to cover the anastomotic site. The sponge was placed transanally intraoperatively to fully cover the anastomotic site. Particular attention was paid to positioning the proximal end of the sponge approximately 2 cm above the anastomosis to ensure adequate coverage and effective drainage. Correct placement was confirmed by digital palpation and endoscopic inspection. Correct placement was confirmed by digital palpation and endoscopic inspection. Continuous negative pressure of approximately 150–200 mbar was applied.

Sponge exchanges were performed at intervals of 3–6 days. During each exchange, the anastomosis was assessed endoscopically for integrity, signs of insufficiency, bleeding, or mucosal abnormalities. Termination of prophylactic pEVAT was guided by intact anastomotic integrity and a healthy-appearing mucosa. In selected cases, preservation of negative pressure was challenged by proximity of the sponge to the anal verge; this was managed by temporary sealing of the anal opening with medical adhesive tape.

### Definition and assessment of anastomotic leakage

Anastomotic leakage was classified according to established criteria (Grades A–C) [15]. Routine imaging to actively screen for asymptomatic Grade A leakage was not performed in the absence of clinical or laboratory signs suggestive of an anastomotic complication.

### Assessment and management of anastomotic stenosis

Anastomotic stenosis was assessed during routine preoperative evaluation for stoma reversal, typically no earlier than three months after the index procedure. All patients scheduled for stoma reversal underwent distal limb contrast radiography as part of the preoperative evaluation to assess anastomotic integrity and exclude occult leakage or fistula. In suspected stenosis, further evaluation included clinical examination and flexible endoscopy. In case of stenosis, endoscopic balloon dilation was preferred over manual dilation due to better patient tolerance and the ability to perform controlled luminal expansion with simultaneous visual assessment of the anastomosis.

### Stoma construction and reversal

A protective diverting stoma was constructed regularly according to German guidelines. The choice between ileostomy and colostomy was individualized based on patient characteristics and bowel anatomy. Stoma reversal was planned following confirmation of anastomotic integrity, generally no earlier than three months after the primary procedure.

### Statistical analysis

Statistical analyses were performed using SPSS (version 25; IBM Corp., Armonk, NY, USA). Continuous variables are presented as mean ± standard deviation or median with range, as appropriate, and categorical variables as counts and percentages. Risk estimation analyses represent unadjusted associations between treatment group and outcome. Given the limited number of anastomotic leakage events, multivariable logistic regression models were intentionally restricted to avoid overfitting. Adjustment was performed using propensity score as a single covariate. A p-value < 0.05 was considered statistically significant.

**Fig. 1:**
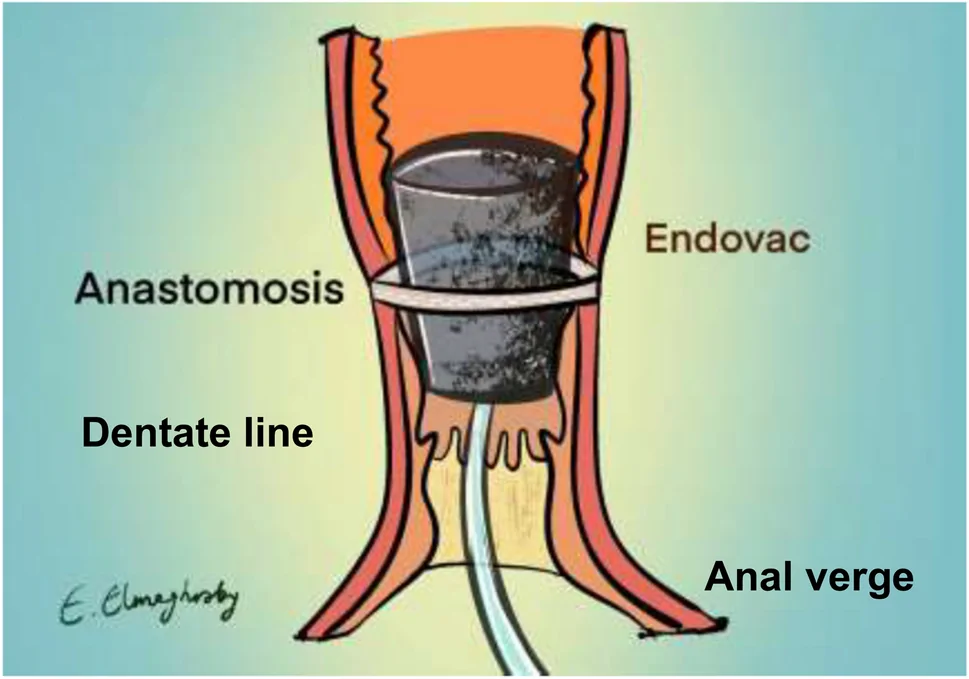
Placement of Prophylactic Endoluminal Vacuum-assisted Therapy (pEVAT)

The authors did not receive financial support from any organisation for the submitted work (Fig. 1).

## Results

**Fig. 2:**
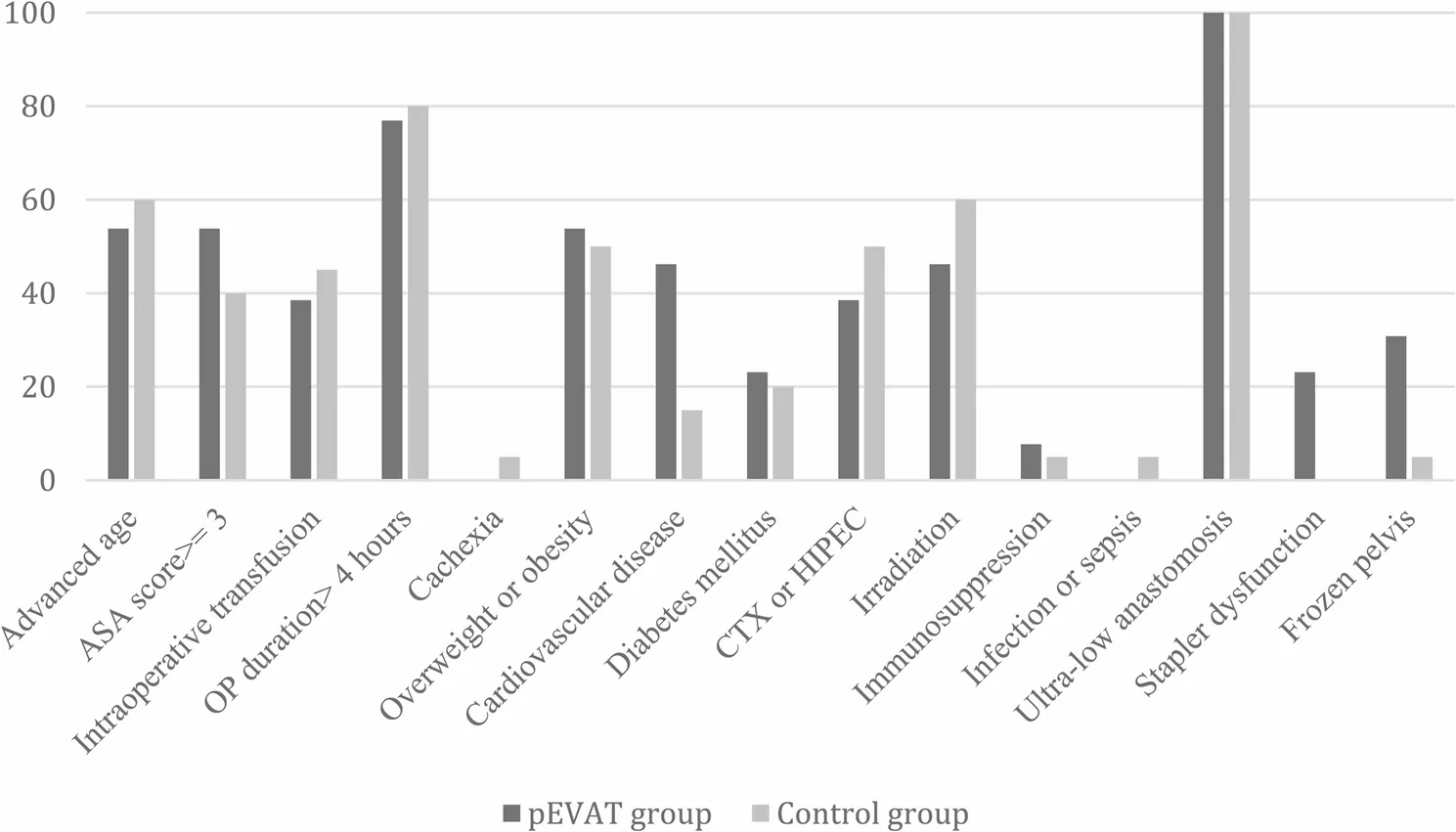
Distribution of risk factors across both groups. pEVAT Prophylactic endoluminal vacuum-assisted therapy, SD Standard deviation, BMI Body-mass-index, ASA American society of Anesthesiologists, CTX: Chemotherapy, HIPEC: Hyperthermic, intraperitoneal chemotherapy

**Fig. 3:**
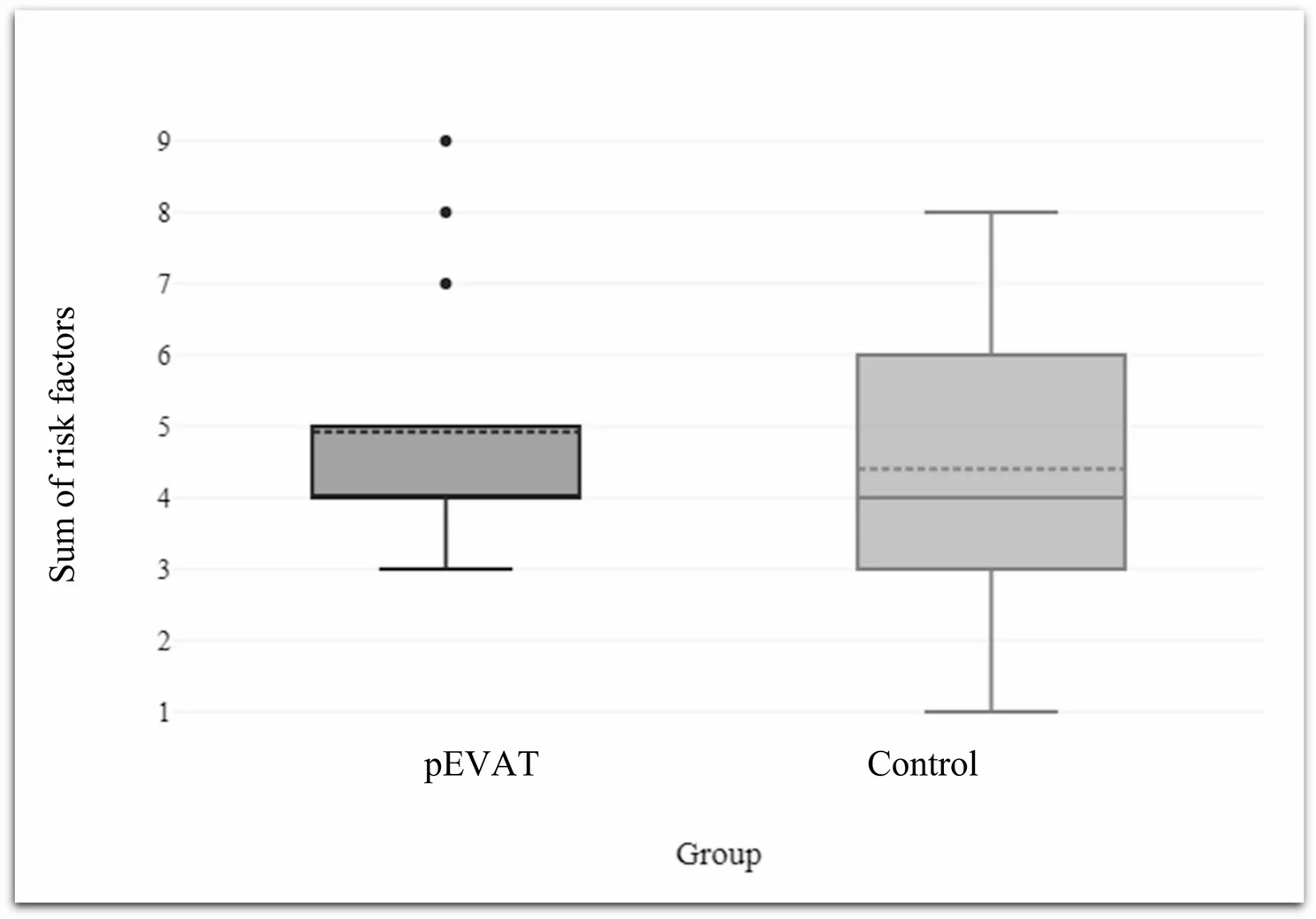
Boxplot of the sum of risk factors in both groups

Between 1/2016 and 12/2020, 33 patients received an uLAR with a diverting stoma for colorectal cancer. pEVAT was applied in 13 patients whereas 20 patients served as a control group without pEVAT. The pEVAT group comprised 9 (69.2%) male and 4 (30.8%) female patients, while the control group included 13 (62.5%) male and 7 (37.5%) female patients. Mean age and BMI were comparable between both groups (Table 1). Both groups had a similar burden of risk factors, including ASA score ≥ 3, cardiovascular disease, prior radiation or chemotherapy, and extended operative time > 4 h (Fig. 2). The mean number of additional risk factors per patient was 4.9 in the pEVAT group and 4.4 in the control group (Fig. 3). The mean distance of the anastomosis from anal verge was also comparable across both groups (3.4 cm in the pEVAT group vs. 3.5 cm in the control group).

In the pEVAT group, only one patient (7.7%) developed a Grade B AL, successfully treated with EVAT and CT-guided drainage. In contrast, six patients (30%) in the control group experienced AL—three Grade B and three Grade C leakages (Table 2). The Grade C patients required reoperation, including one Hartmann’s procedure and one abdominoperineal resection. Complete healing (100%) was achieved in the pEVAT patient, while two control patients suffered persistent complications (10%). However, the difference between the two groups was not significant (*p* = 0.12).

**Table 2:** Frequency of anastomotic leak in both groups. Grade A: radiological diagnosis in an asymptomatic patient, Grade B: anastomotic leak requiring active therapeutic intervention without re-laparotomy, Grade C: anastomotic leak with peritonitis requiring a relaparotomy

Anastomotic leak					
	No	Yes			
		Total	Grade of anastomotic leak		
			Grade *A*	Grade *B*	Grade *C*
pEVAT n (%) *n* = 13	12 (92.3)	1 (7.7)	0 (0.0)	1 (7.7)	0 (0.0)
Control n (%) *n* = 20	14 (70)	6 (30)	0 (0.0)	3 (15)	3 (15)

Anastomotic stenosis occurred in 1 of 13 pEVAT patients (7.7%) and in 7 of 20 control patients (35%) (Table 3). All cases were managed with endoscopic balloon dilation. There was no significant difference between the two groups (*p* = 0.15). Stoma reversal was successfully performed in 76.9% of the pEVAT group and 55% of the control group (*p* = 0.71).

**Table 3:** Other postoperative complications

		pEVAT *n* = 13	Control *n* = 20	*p-value*
Complication n (%)	No	5 (38.5)	8 (40.0)	*0*,*93*
	Yes	8 (61.5)	12 (60.0)	
Surgical site infection n (%)	No	8 (61.5)	14 (70.0)	
	Yes	5 (38.5)	6 (30.0)	*0*,*61*
Bleeding n (%)	No	11 (92.3)	20 (100.0)	
	Yes	1 (7.7)	0 (0.0)	*0*,*07*
Sepsis n (%)	No	13 (100.0)	19 (95.0)	
	Yes	0 (0.0)	1 (5.0)	*0*,*41*
Fistula formation n (%)	No	13 (100.0)	19 (95.0)	
	Yes	0 (0.0)	1 (5.0)	*0*,*41*
Stenosis n (%)	No	9 (69.2)	8 (40.0)	
	Yes	1 (7.7)	7 (35.0)	*0*,*15*
	N/A	3 (23.1)	5 (25.0)	

Mean hospital stay was 26 days in the pEVAT group versus 36 days in the control group. In the pEVAT group, one patient experienced bleeding at the anastomosis site, which was surgically resolved with transanal oversewing of the involved vessel stump. No mortality or fistula formation was observed in either group. Surgical site infection occurred in 38.5% of pEVAT patients and 30% of controls (Table 3).

When comparing both groups and estimating the risk of complications, unadjusted risk estimation demonstrated that the odds of an anastomotic leak in the pEVAT group after uLAR were lower than in the control group (unadjusted OR 0.26; 95% CI 0.04–1.89) (Table 4). In multivariable logistic regression adjusted for propensity score, no independent association between pEVAT and anastomotic leakage was observed (adjusted OR 1.16; 95% CI 0.05–27.03).

**Table 4:** Risk estimation via Odd Ratio (OR) for anastomotic leak, stenosis and stoma reversal

Risk estimation	
Anastomotic leak OR (95% CI)	0.256 (0.035–1.892)
N of valid cases	33
Anastomotic stenosis OR (95% CI)	0.214 (0.031–1.486)
N of valid cases	25
Successful stoma reversal OR (95% CI)	1.288 (0.835–1.985)
N of valid cases	29

The odds of anastomotic stenosis without pEVAT were higher compared to patients receiving pEVAT (unadjusted OR 0.21; 95% CI 0.03–1.49). Additionally, the likelihood of successful stoma reversal after pEVAT was higher compared to no pEVAT treatment (unadjusted OR 1.28; 95% CI 0.83–1.98).

## Discussion

Anastomotic leaks have an enormous negative impact on patients with rectal cancer undergoing ultra-low anterior resection with primary anastomosis. They increase morbidity and mortality, substantially impair quality of life through scarring and stenosis, and may negatively affect stoma reversal. Moreover, anastomotic leakage can delay the initiation of adjuvant chemotherapy, potentially compromising oncological outcomes [16, 17].

Therefore, the implementation of prophylactic endoluminal vacuum-assisted therapy (pEVAT) in high-risk ultra-low anterior resections represents a novel strategy in colorectal surgery. The concept of pEVAT was inspired by the established prophylactic use of negative pressure wound therapy (NPWT) in contaminated or high-risk wounds in abdominal surgery to reduce surgical site infections [18, 19]. EVAT is widely used for the treatment of established anastomotic leakage; however, its prophylactic application in colorectal surgery remains limited. We previously reported a first series of patients receiving pEVAT after uLAR for a variety of etiologies, demonstrating technical feasibility and encouraging clinical outcomes [14]. Existing literature also shows high success rates for therapeutic EVAT, particularly when applied early after diagnosis of anastomotic leakage [9, 20, 21]. These observations provided the rationale for intraoperative implementation of pEVAT to support anastomotic healing in selected high-risk situations.

In the present study, pEVAT did not demonstrate a statistically significant reduction in anastomotic leakage compared with existing literature for low anterior resection [2, 22, 23]. However, a lower incidence of leakage was observed in the pEVAT group compared with the internal control cohort (7.7% vs. 30%). This difference must be interpreted cautiously, as both study groups consisted exclusively of patients with ultra-low anastomoses and multiple cumulative risk factors for leakage. Rullier et al. reported a 6.5-fold increase in anastomotic leakage rates when the anastomosis is located ≤ 5 cm from the anal verge [3]. The combination of ultra-low anastomotic level and additional risk factors likely explains the relatively high leakage rate observed in the control group, and residual confounding cannot be excluded. Accordingly, the present findings suggest a potential protective signal rather than a proven effect of pEVAT.

Notably, the only patient who developed an anastomotic leak in the pEVAT group was managed by continuation of sponge therapy as therapeutic EVAT, resulting in complete healing without the need for surgical revision such as Hartmann’s procedure. This observation suggests that prophylactic pEVAT may also facilitate early management of leakage when it occurs, potentially mitigating its clinical consequences and decreasing the time toward healing of the anastomosis, although this hypothesis requires confirmation in prospective studies. The mean duration of pEVAT was substantially shorter (8.7 days) compared with reported EVAT treatment durations for established anastomotic leakage (median 47 days), with fewer sponge changes required. While this may have contributed to shorter hospital stays and earlier recovery, length of hospitalization in this cohort was influenced by multiple additional factors unrelated to anastomotic healing. These included postoperative medical complications such as pneumonia, urinary tract infections, or thromboembolic events, as well as surgical site infections requiring prolonged wound management, including vacuum-assisted wound therapy in selected cases. In addition, delays in transfer to rehabilitation facilities, step-down units, or discharge with ambulatory nursing support frequently contributed to extended hospital stay. Therefore, differences in length of stay should be interpreted cautiously and descriptively, as this retrospective analysis was not designed to assess recovery time or resource utilization. Formal cost-effectiveness analyses were beyond the scope of this investigation.

Complications directly attributable to pEVAT were infrequent and included one case of bleeding and one anastomotic stenosis. The bleeding may have been related to direct contact between the polyurethane sponge and a small, exposed vessel stump at the stapled anastomosis under continuous negative pressure. Polyurethane sponges should generally not be placed in direct contact with exposed vessels due to the risk of erosion bleeding. Importantly, this rare complication was easily managed and no further bleeding events occurred, supporting the overall safety of pEVAT when careful inspection of the anastomosis is performed prior to sponge placement, although conclusions here are limited by the small sample size. The lower rate of anastomotic stenosis observed in the pEVAT group (7.7% vs. 35%) is an unexpected but encouraging finding when compared with existing literature [24–26]. Although concerns have been raised that negative pressure may promote fibrosis and stricture formation, this was not observed in the present study. Longer treatment duration and more severe anastomotic leakage in the control group may partly explain the higher stenosis rate, but this remains speculative.

Stoma reversal rates were higher in the pEVAT group (76.9% vs. 55%), suggesting that a greater proportion of patients were able to restore intestinal continuity. Importantly, prophylactic pEVAT did not appear to adversely affect stoma reversal rates, which are comparable to those reported in the literature [27, 28]. While no statistically significant differences were observed, the consistency of trends across multiple clinically relevant endpoints is noteworthy but must be interpreted in the context of limited statistical power.

Despite these observations, this study has several limitations. The small sample size and limited number of outcome events restrict statistical precision and preclude definitive conclusions regarding efficacy. As a retrospective, non-randomized analysis, the risk of selection bias and residual confounding is substantial, particularly as the decision to apply pEVAT was based on surgeon judgment. Additionally, variation in pEVAT duration and application may have influenced outcomes. A small proportion of patients were lost to follow-up, limiting assessment of long-term endpoints such as anastomotic stenosis and stoma reversal. Larger, prospective, and ideally randomized studies with standardized follow-up protocols are therefore required to clarify the clinical value of pEVAT, identify patients most likely to benefit, and evaluate patient-centred and economic outcomes.

## Conclusion

In summary, prophylactic endoluminal vacuum-assisted therapy is a feasible and safe approach that may have a potential protective role against Anastomotic leak in selected high-risk patients with colorectal cancer undergoing ultra-low colorectal anastomosis. However, given the retrospective design, limited sample size, and absence of statistical significance, definitive conclusions regarding efficacy cannot be drawn, and larger prospective and ideally randomized studies are required to clarify its clinical role and establish standardized treatment protocols.

## Data Availability

The datasets generated and/or analyzed during the current study are not publicly available but are available from the corresponding author upon reasonable request.
